# Beyond the numbers: Impact of obesity on obstetric anal sphincter injury (OASI) outcomes in women

**DOI:** 10.1002/ijgo.15981

**Published:** 2024-10-21

**Authors:** Reut Rotem, Daniel Galvin, Kate McCormack, Orfhlaith E. O'Sullivan, Deirdre Hayes‐Ryan

**Affiliations:** ^1^ Department of Urogynecology Cork University Maternity Hospital Cork Ireland; ^2^ Department of Obstetrics and Gynecology, Shaare Zedek Medical Center Affiliated with the Hebrew University School of Medicine Jerusalem Israel; ^3^ Department of Obstetrics and Gynecology Cork University Maternity Hospital Cork Ireland

**Keywords:** anatomical defects, body mass index (BMI), endoanal manometry, endoanal ultrasound, OASI clinic, obesity, obstetric anal sphincter injury (OASI)

## Abstract

**Objective:**

To compare the risk profiles, anatomical, and functional outcomes between obese and non‐obese women who experienced obstetric anal sphincter injury (OASI).

**Methods:**

A retrospective electronic database study was conducted at Cork University Maternity Hospital (CUMH). Women with missing data/repairs conducted outside CUMH were excluded. Participants were categorized into obese (BMI ≥30 kg/m^2^) and non‐obese (BMI <30 kg/m^2^) groups. Primary measure was a composite adverse outcome assessed 6 months post‐delivery, including one or more of the following: resting pressure <40 mmHg, squeezing pressure <100 mmHg, defects in the internal and/or external anal sphincter. Statistical analyses were performed using SPSS version 28.

**Results:**

Among the 349 women included in the study, 285 (81.7%) had a BMI <30 kg/m^2^ and 64 (18.3%) had a BMI ≥30 kg/m^2^. Gestational diabetes was significantly higher in obese women. No significant differences were observed in newborn weight or mode of delivery. The majority of tears were classified as grade 3B in both groups. Attendance rates at the OASI clinic did not differ between the groups. Among those attending, no statistical differences were noted in manometry results, which were reduced in both groups. Rates of internal anal sphincter defects were lower in the obese group (7.0% vs 15.6%, *P* = 0.15) and external anal sphincter defects were significantly lower in obese women (0% vs 9.1%, *P* = 0.04). No difference was found in the rates of composite adverse outcomes between the groups.

**Conclusion:**

Functional outcomes and manometry results did not differ, but non‐obese women had higher rates of anatomical defects in OASI, requiring further study.

## Synopsis

Obese and non‐obese women had similar functional outcomes after obstetric anal sphincter injury, but objective assessments showed worse outcomes in non‐obese women.

## 1 INTRODUCTION

Obstetric anal sphincter injuries (OASI) are severe complications during vaginal deliveries, involving damage to the anal sphincter complex.^1^ Globally, the incidence of OASI is approximately 11%^2^ among vaginal births, though this rate can vary widely depending on the population and healthcare practices. In Ireland, the incidence is notably lower, around 1.6%^3^ of deliveries. These injuries can have devastating short‐ and long‐term consequences, including anal incontinence, chronic pain and significant impacts on quality of life and mental health.^1^

Obesity, defined as a body mass index (BMI, calculated as weight in kilograms divided by the square of height in meters) of 30 or greater, has become a growing global health concern.^4^ The prevalence of obesity has been increasing steadily, with current statistics indicating that a substantial proportion of the adult population is affected.^4^ This rise is particularly concerning among pregnant women, where obesity is associated with a range of adverse maternal and fetal outcomes. The increasing number of obese pregnant women underscores the need to understand the implications of obesity on pregnancy and childbirth outcomes.^5^ Obesity is associated with several risk factors that increase the likelihood of OASI.^6^ These include gestational diabetes, macrosomia (large‐for‐gestational‐age babies), higher rates of instrumental deliveries such as forceps and vacuum extractions, shoulder dystocia and prolonged labor.^6^ Each of these factors independently raises the risk of perineal trauma during childbirth, and their prevalence is higher among obese women, compounding the risk of OASI.^2^

Contrary to the expectation that obesity would increase the risk of OASI due to the aforementioned factors, most studies to date have found that obesity is associated with lower rates of OASI.^7^, ^8^, ^9^ This inverse relationship appears to be dose‐dependent, with higher BMI correlating with a decreased incidence of OASI.^7^ The reasons for this counterintuitive finding are not fully understood and warrant further investigation.

While there is considerable research on the incidence and immediate outcomes of OASI in relation to obesity, data on the long‐term outcomes for obese women are scarce. The present study aimed to address this gap by evaluating medium‐term outcomes (6 months post‐delivery) of OASI in obese women compared to their non‐obese counterparts. Understanding these outcomes is crucial for developing targeted interventions and improving clinical practices and the aim of the present study was to contribute to existing knowledge and inform future guidelines for managing and preventing OASI, especially with rising obesity rates.

## 2 MATERIALS AND METHODS

### 2.1 Study design

A retrospective study was conducted at Cork University Maternity Hospital (CUMH), a prominent tertiary university teaching maternity hospital with approximately 7000 deliveries annually. The study encompassed all deliveries complicated by obstetric anal sphincter injuries (OASI) that were repaired at CUMH between 2018 and 2023. To assess whether obese women possess a distinct risk profile for OASI and endure worse anatomical and functional outcomes, we compared obese women (BMI ≥30) to non‐obese women (BMI <30), with BMI calculated as the weight at delivery.

### 2.2 Data collection

Data were obtained from the electronic database of CUMH (Maternal & Newborn Clinical Management System [MNCMS] electronic health care records), which is continuously updated in real‐time by a team of midwives and obstetricians throughout the delivery process and postpartum period. This study integrated two sources of data: one detailing deliveries at the hospital and another from follow‐up visits at the OASI clinic. This approach allowed us to consider women who delivered at CUMH and later attended the OASI clinic, as well as those who attended the OASI clinic without having delivered at CUMH. Women who had deliveries >24 weeks of gestation, suffered an OASI, and were repaired at CUMH and later seen in our OASI clinic were included in the study. Exclusion criteria encompassed women with missing data or those who underwent repair procedures outside of CUMH, even if they were later seen in the OASI clinic.

### 2.3 OASI repair protocol

In accordance with hospital protocol, OASI repairs are usually performed in the operating theater under optimal anesthesia conditions to ensure the best surgical outcomes, following formal written patient consent. Repairs may be conducted by either a non‐consultant hospital doctor (NCHD), who has completed formal appropriately recognized training, or by a consultant. Prophylactic intravenous antibiotics are administered prior to the procedure. Surgical techniques employed include both end‐to‐end and overlap suturing methods. Internal anal sphincter (IAS) tears are usually sutured end‐to‐end, while external anal sphincter (EAS) tears are mostly sutured using the overlap method, except in cases of 3A tears where end‐to‐end suturing might be considered. Patients are typically debriefed by the surgical team during hospitalization and discharged with a five‐day course of oral prophylactic antibiotics (amoxicillin/clavulanic acid and metronidazole).^10^ They are advised to avoid constipation and to use stool softeners as necessary. All patients are referred for pelvic floor physiotherapy after delivery and reviewed by a physiotherapist prior to discharge. During this review, patients receive comprehensive information and education regarding pelvic floor health and postnatal recovery. A follow‐up phone call is conducted by the physiotherapist at 6 weeks to assess for any ongoing symptoms. If symptoms are present, the patients are scheduled for an in‐person consultation; if not, they are discharged from the physiotherapy follow‐up. Six weeks post‐delivery, patients are invited for a post‐natal review, where initial symptom assessments and physical examinations are conducted by a certified consultant. Four to six months post‐delivery, patients are invited to attend the local OASI clinic, a specialized clinic overseen by a certified consultant with specific training in this area. Upon arrival at the clinic, their medical and obstetrical history is verified, and the St. Marks score is calculated to assess sphincter compromise and the severity of fecal/flatal incontinence, if present. The St. Marks score,^11^ also known as the Vaizey score, ranges from 0 to 24, with a score of 0 indicating perfect continence and a score of 24 indicating complete incontinence. This scoring system evaluates several factors, including the frequency of incontinence episodes, alterations in lifestyle, and the use of protective garments.^11^ The score is a validated and widely used tool for evaluating anal sphincter function and guiding treatment decisions.^11^ With patient consent, a gentle physical examination, including para‐vaginal (PV) and para‐rectal (PR) examinations, is carried out. This is followed by endo‐anal ultrasound (EUS) and endoanal manometry to identify anatomical defects in the sphincter and to objectively measure the resting and squeeze pressures of the anal sphincter, indicating the functionality of the IAS and EAS, respectively. Based on each patient's symptoms and objective assessments, detailed consultations regarding subsequent delivery options are provided, and referrals to colorectal surgeons are made when necessary.

### 2.4 Outcome measures

The primary outcome of the study was composite adverse outcomes at 6 months post‐delivery in the study groups, defined as having one or more of the following: resting pressure <40 mmHg, squeezing pressure <100 mmHg, defect to the IAS, or defect to the EAS. These measures were selected because they are objective and provide a reliable assessment of sphincter function. Secondary outcomes included various risk factors in both study groups.

### 2.5 Statistical analysis

The characteristics of the study population are described using proportions for nominal variables, means ± standard deviations (SD) for continuous variables with normal distribution, and medians with interquartile ranges (IQR) for continuous variables without normal distribution, as appropriate. Categorical variables were compared using the chi‐square test or Fisher exact test, and continuous variables were analyzed using the unpaired student's *t*‐test or Mann–Whitney test, as appropriate. A *P* value of <0.05 was considered statistically significant. All statistical tests were two‐sided and conducted using SPSS software (version 28; IBM, Armonk, NY). Ethical approval for this study was received from the Clinical Research Ethics Committee of the Cork Teaching Hospitals ECM. Data for the study were obtained from electronic health care records, and all patient information was de‐identified. Consequently, direct participation of patients was not required, and written informed consent was waived.

## 3 RESULTS

During the study period (2018–2023), there were a total of 23 461 vaginal deliveries at CUMH, of which 380 were complicated by OASI, representing a rate of 1.6%. After applying the inclusion and exclusion criteria, 349 cases were included in the study, with 285 (81.7%) women having a BMI <30 and the remaining 64 (18.3%) being classified as obese with a BMI ≥30.

Table 1 displays maternal and pregnancy characteristics, which were overall comparable between the groups, except for gestational diabetes, which was significantly higher among obese women (23.4% vs 5.3%, *P* < 0.01). The majority of women were nulliparous, with comparable rates between the groups.

**TABLE 1:** Maternal and pregnancy characteristics.

Variable	BMI <30 *N* = 285	BMI ≥30 *N* = 64	*P* value
Maternal age, year, mean ± SD	32.20 ± 4.68	32.48 ± 4.81	0.66
Ethnicity, *N* (%)			
Caucasian	232 (81.4%)	52 (81.2%)	0.10
Asian	34 (11.9%)	6 (9.4%)	
Other	53 (6.7%)	6 (9.4%)	
Parity, median (IQR)	0 (0–1)	0 (0–1)	0.52
Nulliparity, *N* (%)	196 (68.8%)	46 (71.9%)	0.62
Private obstetric care, *N* (%)	29 (10.2%)	2 (3.1%)	0.07
Gestational diabetes mellitus, *N* (%)	15 (5.3%)	15 (23.4%)	<0.01

*Note*: BMI, calculated as weight in kilograms divided by the square of height in meters.

Abbreviations: BMI, body mass index; IQR, interquartile range; SD, standard deviation.

Table 2 presents delivery characteristics of the study groups. Gestational age at delivery and newborn weight did not differ significantly between the groups. Rates of labor augmentation were higher among obese women, though not significantly (18.8% vs 12.3%, *P* = 0.17). Mode of delivery and fetal presentation at delivery were comparable between the groups.

**TABLE 2:** Delivery characteristics.

Variable	BMI <30 *N* = 285	BMI ≥30 *N* = 64	*P* value
Place of delivery, *N* (%)			
Hospital	261 (91.6%)	54 (84.4%)	0.11
Born before arrival	22 (7.7%)	8 (12.5%)	
Planned home birth	2 (0.7%)	2 (3.1%)	
Gestational age at delivery, weeks, mean ± SD	39.46 ± 1.35	39.52 ± 1.56	0.33
Newborn weight, grams, mean ± SD	3620.86 ± 478.12	3642.48 ± 494.26	0.75
Mode of delivery, *N* (%)			
Spontaneous vaginal delivery	145 (50.9%)	36 (56.3%)	0.72
Forceps Vacuum extraction	62 (21.8%) 78 (27.4%)	13 (20.3%) 15 (23.4%)	
Labor induction, *N* (%)	128 (44.9%)	29 (45.3%)	0.95
Labor augmentation, *N* (%)	35 (12.3%)	12 (18.8%)	0.17
Second stage of labor, minutes, median (IQR)	72 (31–121)	46 (20–104)	0.08
Fetal position at delivery, *N* (%)			
OP	36 (12.6%)	8 (12.5%)	0.30
OT	16 (5.6%)	7 (10.9%)	
Shoulder dystocia, *N* (%)	18 (6.3%)	3 (4.7%)	0.62
Episiotomy, *N* (%)	148 (51.9%)	27 (42.2%)	0.16

*Note*: BMI, calculated as weight in kilograms divided by the square of height in meters.

Abbreviations: BMI, body mass index; IQR, interquartile range; OASI, obstetric anal sphincter injury; SD, standard deviation.

Table 3 presents OASI repair characteristics and follow up. In both groups, the majority of OASI cases were classified as grade 3B tears followed by grade 3A tears. Most of these were repaired by NCHD using the end‐to‐end technique, with no significant differences observed between the groups. Attendance rates at the local OASI clinic review at 6 months post‐delivery did not differ between the groups, with rates of 67.2% (43/64) for obese women and 65.7% (186/285) for non‐obese women.

**TABLE 3:** OASI repair characteristics and follow‐up.

Variable	BMI <30 *N* = 285	BMI ≥30 *N* = 64	*P* value
Grade of OASI, *N* (%)			
3A	104 (36.5%)	26 (40.6%)	0.89
3B	139 (48.8%)	29 (43.5%)	
3C	30 (10.5)	6 (9.4%)	
4	10 (3.5%)	3 (4.7%)	
Buttonhole	2 (0.8%)	0 (0.0%)	
Operator repairing the OASI, *N* (%)			
Non‐consultant hospital doctor	233 (81.5%)	57 (89.1%)	0.14
Consultant	52 (18.5%)	7 (10.9%)	
Suture technique, *N* (%)			
End to end	214 (77.3%)	45 (76.3%)	0.48
Overlap	71 (22.7%)	19 (23.7%)	
Estimated blood loss, mL, median (IQR)	400 (250–700)	400 (250–800)	0.57
Wound infection, *N* (%)	19 (6.7%)	2 (3.1%)	0.27
Attendance to 6‐week post‐natal review, *N* (%)	202 (70.9%)	39 (60.3%)	0.45
Perineal clinic review at 6 months, *N* (%)	186 (65.7%)	43 (67.2%)	0.82

*Note*: BMI, calculated as weight in kilograms divided by the square of height in meters.

Abbreviations: BMI, body mass index; IQR, interquartile range; OASI, obstetric anal sphincter injury.

Outcomes and local OASI clinic assessments of the women who attended the clinic are presented in Table 4. Fecal symptoms were reported by 18.6% of obese women and 25.8% of non‐obese women, with no significant difference between the two groups (*P* = 0.31). Overall, St. Marks scores for both groups were low and comparable. Endoanal manometry results indicated that resting pressure of the internal anal sphincter was significantly higher in obese women (37 vs 31 mmHg, *P* < 0.01). Similarly, squeezing pressure of the external anal sphincter was higher in obese women (65 vs 56 mmHg, *P* = 0.03). While the rate of sonographic defects in the internal anal sphincter was lower in the obese group (7.0% vs 15.6%), this difference did not reach statistical significance (*P* = 0.15). However, there were no cases of sonographic defects in the external anal sphincter in the obese group compared to 17 cases (9.1%) in the non‐obese group (*P* = 0.04). Rates of composite adverse outcomes and referrals to colorectal surgeons did not differ significantly between the groups.

**TABLE 4:** Outcomes and perineal clinic assessments.

Variable	BMI <30 *N* = 186	BMI ≥30 *N* = 43	*P* value
Fecal symptoms, *N* (%)	48 (25.8%)	8 (18.6%)	0.31
St. Marks score, median (IQR)	0 (0–2)	0 (0–1)	0.17
Rest pressure, median (IQR)	31 (25–39)	37 (30–43)	<0.01
Squeeze pressure, median (IQR)	56 (46–72)	65 (54–73)	0.03
EUS defect to internal anal sphincter, *N* (%)	29 (15.6%)	3 (7.0%)	0.15
EUS defect to external anal sphincter, *N* (%)	17 (9.1%)	0 (0.0%)	0.04
Patient referred to colorectal specialist, *N* (%)	9 (4.8%)	1 (2.3%)	0.47
Composite adverse outcomes *N* (%)	174 (93.5%)	39 (84.7%)	0.50

*Note*: BMI, calculated as weight in kilograms divided by the square of height in meters. EUS composite adverse outcome; having one or more of the following: Rest pressure <40 mmHg, squeeze pressure <100 mmHg, defect to internal anal sphincter, defect to external anal sphincter.

Abbreviations: BMI, body mass index; EUS, endo‐anal ultrasound; IQR, interquartile range.

## 4 DISCUSSION

Our study examined the medium‐term outcomes of OASI in obese and non‐obese women and demonstrated that there was no difference in functional outcomes, as reflected by fecal symptoms and St. Mark's score between the groups. Additionally, endoanal manometry results, despite not achieving statistical significance, were better among the obese. Anatomically, sonographic defects to both sphincters were lower in the obese group.

The overall rate of OASI observed in our study was 1.6%, which is relatively low but aligns with previous data indicating a lower incidence of OASI in Ireland compared to global averages.^12^ Additionally, it is worth noting the relatively high prevalence of Asian women in both groups of our study, who are known to be at higher risk for OASI.^13^

The global obesity pandemic is a growing public health crisis, with the WHO estimating that obesity rates have nearly tripled since 1975.^14^ This surge in obesity prevalence has significant long‐term health implications, imposes a substantial financial burden on healthcare systems, and profoundly impacts quality of life.^4^ Among pregnant women, obesity exacerbates risks, leading to complications such as gestational diabetes, pre‐eclampsia, and higher rates of cesarean delivery.^6^ Despite these risks, the literature states that the incidence of OASI in obese women is actually lower than that of their non‐obese counterparts.^7^, ^8^, ^9^ This counterintuitive finding suggests that obesity may play a protective role against OASI. A possible explanation is that excess adipose tissue in obese women may act as a physical barrier, and softer perineal tissue may provide additional cushioning and protection to the anal sphincter during childbirth.^9^ Although our data did not directly support this hypothesis due to a lack of incidence information, our focus on outcomes revealed that obesity is associated with better outcomes after OASI.

Obesity is known to impair wound healing,^15^ yet our study found that wound infection rates among the obese were not higher and were, in fact, lower, although not statistically significant. This is interesting given the literature linking obesity to wound infections after cesarean sections and other surgical procedures.^16^, ^17^ This might be due to the meticulous postoperative care at our institution, including the use of broad‐spectrum antibiotics and stimulant laxatives as per the Royal College of Obstetrics and Gynecology Green‐Top Guidelines.^10^

In our cohort, manometry results were low among both obese and non‐obese women. However, women with a BMI over 30 had higher resting and squeezing pressures, although this was not statistically significant. EUS showed a higher rate of defects to both the EAS and IAS among the non‐obese group, with no cases of EAS defects in the obese group, representing a statistically significant difference. Despite unfavorable manometry and EUS results in both groups, there was a low rate of persistent symptoms such as fecal or flatal incontinence, as reflected by the St. Mark's score. This suggests a low burden of symptoms in our unit. The poor correlation between objective tests and subjective symptoms has been previously reported in the literature.^18^, ^19^, ^20^ This discrepancy may be due to the complex interplay of factors influencing symptom perception, including individual variations in symptom tolerance, psychological factors, and differences in tissue healing and adaptation. Furthermore, the favorable clinical outcomes in our cohort may indicate overdiagnosis and misclassification of tears,^21^ where vaginal tears are classified as perineal tears, or low‐grade perineal tears are categorized as more severe. These results highlight the importance of comprehensive clinical assessments that extend beyond endoanal manometry alone. It is crucial to consider the patient's overall experience and symptoms to effectively manage and treat OASI.

Across all parameters assessed at our OASI clinic (St. Mark's score, endoanal manometry, and ultrasound assessment of the anal sphincter complex), obese women did not have worse outcomes. This may be attributed to several factors. Hormonal differences in obese women might alter tissue elasticity and repair mechanisms, enhancing recovery and reducing long‐term complications. Nonetheless, these results should not be interpreted as an endorsement of obesity, which remains a serious health concern with significant long‐term implications. Further research is needed to fully understand the relationship between obesity and OASI outcomes, with a focus on developing targeted interventions that support maternal health, including dietary consultation and weight management during pregnancy. Future studies should consider longitudinal data to assess long‐term outcomes beyond 6 months and explore the role of lifestyle and weight management in enhancing recovery and reducing OASI incidence.

Our study had several strengths. It was a large‐scale investigation focusing on women 6 months post‐delivery, providing valuable medium‐term outcome data. All women were treated under standardized protocols and followed up in the same clinic by a board‐certified consultant, reducing potential bias. The diverse population in our study enhances the generalizability of our results. To the best of our knowledge, this is the first study to examine medium‐term outcomes of OASI in obese women, contributing significantly to existing knowledge.

However, there are limitations to our study. The retrospective nature of the present study introduces inherent biases, particularly regarding those who did not attend follow‐up visits. While this poses a limitation, we attempted to mitigate this by adhering to a standardized follow‐up protocol for all women with OASI. Unfortunately, we were unable to calculate the specific incidence of OASI for obese and non‐obese populations, as our dataset lacked the necessary distribution data by BMI. Additionally, we were unable to characterize patients who did not return for follow‐up at the perineal clinic, which may introduce selection bias. However, as clinic attendance rates at 6 months were similar across both groups, we believe that any bias introduced is likely non‐differential. We were also unable to characterize excluded patients due to missing data, and their obesity distribution remains unknown. Additionally, obesity has subclassifications but our sample size was not large enough to allow for subanalysis within these categories, which might have different outcomes. The relatively low rate of OASI in Ireland resulted in a smaller sample size, which may limit the power of our findings. Regarding wound healing, some data pertaining to other risk factors for poor outcomes in this area, such as smoking, were not documented. This could potentially influence the outcomes related to wound healing and infection rates.

In conclusion, our study suggests that obesity may confer a protective effect against unfavorable long‐term outcomes of OASI despite multiple risk factors. These findings challenge existing assumptions about obesity's impact on childbirth‐related injuries and highlight the need for a detailed understanding of the relationship between maternal BMI and OASI outcomes. Further research is needed to elucidate these mechanisms and confirm these results in larger, prospective studies. Understanding these dynamics will be crucial for developing targeted interventions and improving clinical practices for all women, irrespective of their BMI.

## AUTHOR CONTRIBUTIONS

R. Rotem: Protocol development, data collection and management, data analysis, and manuscript writing/editing. D. Galvin: Protocol development, data collection and management, data analysis, manuscript writing/editing. K. McCormack: Data collection and management, manuscript writing/editing. O. E. O'Sullivan: Data collection and management, manuscript writing/editing. D. Hayes‐Ryan: Protocol development, data collection and management, data analysis, manuscript writing/editing.

## FUNDING INFORMATION

No financial support was received for this study.

## CONFLICT OF INTEREST STATEMENT

The authors report no conflict of interest.

## Data Availability

Research data are not shared.
